# Detoxification Metabolic Adaptation of *Bombyx mori* to Artificial Diet and Functional Study of Key Detoxification Gene *BmGSTd2*

**DOI:** 10.3390/insects17030261

**Published:** 2026-02-28

**Authors:** Lijing Liu, Long He, Xin Tang, Qingyou Xia, Ping Zhao

**Affiliations:** 1 Biological Science Research Center, Integrative Science Center of Germplasm Creation in Western China (CHONGQING) Science City, Southwest University, Chongqing 400715, China; liuljzyy@163.com (L.L.); 18202335220@163.com (L.H.);; 2 Chongqing Key Laboratory of Innovative Chinese Medicine and Health Intervention, Chongqing Academy of Chinese Materia Medica, Chongqing University of Chinese Medicine, Chongqing 400065, China; tangxin@cqctcm.edu.cn

**Keywords:** dietary adaptability, detoxification metabolism, *GSTd2*, artificial diet, silkworm (*Bombyx mori* L.)

## Abstract

The exceptional adaptability of insects to diverse food sources is central to their survival and evolutionary success. However, the molecular mechanisms underlying this rapid adaptation remain largely uncharacterized. In this study, adaptive phenotypic, transcriptomic, and metabolomic differences in silkworms fed mulberry leaves versus artificial diets were investigated. The results showed that dietary changes induced enrichment of multiple detoxification pathways in the fat body, midgut, and Malpighian tubules, accompanied by significant accumulation of secondary metabolites and xenobiotics such as flavonoids, terpenoids and saponins in these tissues. Stimulation experiments with nine upregulated metabolites in silkworm BmE cells revealed that most metabolites inhibited cell viability and induced detoxification genes such as *GST*, *UGT* and *CYP* upregulated, with flavonoids like genistein and daidzin exhibiting obvious inductive effects. Among the upregulated genes, *GSTd2* frequently responded and was significantly upregulated in artificial diet-fed silkworms. Notably, overexpressing *GSTd2* in BmE cells enhanced cell tolerance to genistein and daidzin. Furthermore, silkworms overexpressing *GSTd2* showed higher flavonoid tolerance and better adaptability to artificial diets. In conclusion, this study provides valuable genetic targets for improving silkworm rearing efficiency on artificial diets, providing reference to optimize feed formulations and theoretical basis for understanding metabolic adaptation mechanisms to artificial diets in silkworms.

## Simple Summary

This study aims to explore the adaptive mechanisms underlying the silkworm’s (*Bombyx mori*) transition from a natural mulberry leaf diet to an artificial diet. The results showed that dietary shift induced the accumulation of specific plant-derived compounds (e.g., flavonoids) in key detoxification organs of silkworms—including the fat body, midgut, and Malpighian tubules—while activating multiple detoxification pathways within these tissues. Experimental analysis showed that these compounds, particularly the flavonoids genistein and daidzin, exhibit cytotoxicity and can trigger the transcriptional activation of detoxification-related genes. Among these genes, *GSTd2* emerged as a critical mediator: elevated expression of *GSTd2* in BmE cells significantly enhanced cellular tolerance to these harmful flavonoids. Furthermore, silkworms with systemic overexpression of *GSTd2* displayed markedly improved adaptability to the artificial diet. Collectively, this study elucidates the molecular basis of dietary adaptation in insects, providing valuable insights for optimizing artificial feed formulations and enhancing the efficiency of silkworm rearing.

## 1. Introduction

Insects, as the group with the highest species diversity on Earth, have their rapid adaptability to complex food environments as a core driver of long-term evolution [1]. This adaptability is not only manifested at the morphological and behavioral levels but more fundamentally relies on metabolic remodeling at the molecular level—particularly the efficient recognition and metabolism of plant secondary metabolites and xenobiotics by detoxification enzyme systems [2,3]. Silkworm (*Bombyx mori* L.) is an important economic insect and a classic model for studying lepidoptera insects as well [4]. Prolonged artificial selection has led to its preference for mulberry leaves [5,6], resulting in its poorly adapted to non-mulberry food sources like artificial diets. Silkworms reared on artificial diets exhibit low feeding efficiency and asynchronous development [7,8]. This dietary specialization also limits the large-scale breeding and industrialization promotion of silkworm rearing with artificial diets.

Although artificial diets for silkworms contain mulberry leaf components, they also include defatted soybean powder as a key protein source, cornmeal as an important energy source, and other food additives such as propyl gallate [9,10]. Among these, soybean powder and cornmeal are rich in secondary metabolites and other anti-nutritional factors [11], such as flavonoids including genistein, daidzin, glycitin [12], and soy saponins including type A and B [13,14]. In addition, there are triterpenoids, tetraterpenes [15,16], tannins [17], soybean oligosaccharides [18], etc. Although mulberry leaves also contain flavonoids, terpenoids, and tannins, the specific compounds differ from those in artificial diets [19,20]. The terpenoids in soybean powder are mainly triterpenoid saponins, whereas mulberry leaves are rich in volatile monoterpenoids, sesquiterpenoids, as well as triterpenoid alcohols and triterpenoid acids [21,22]. Therefore, exploring the metabolic adaptation mechanisms of silkworms to artificial diets and their abundant secondary metabolites and xenobiotics is particularly important.

The research on insect detoxification mechanisms has predominantly focused on agricultural pests and their metabolism of pesticides [23,24,25], with core pathways including CYP (cytochrome P450)-mediated oxidation and dealkylation, GST (glutathione S-transferase)-catalyzed conjugation reactions with glutathione, and UGT (uridine diphosphate glucuronosyltransferase)-involved conjugative inactivation with UDP-glucuronic acid and UDP-glucose [26,27]. However, lepidoptera insect responses to plant secondary metabolites are highly species-specific. For example, *Helicoverpa armigera* and *Heliothis virescens* enhances its detoxification tolerance to the sesquiterpene dimer gossypol in cotton through glycosylation mediated by *UGT41B3* and *UGT40D1* genes [28]. The genome data of the fall armyworm *Spodoptera frugiperda* show that the *P450* gene family has significantly expanded, which may endow it with stronger detoxification ability against plant volatiles [29]. In the study of *Spodoptera litura*, when larvae were exposed to maize-derived HIPVs (herbivore-induced plant volatiles) or defensive secondary metabolites DIMBOA (2,4-dihydroxy-7-methoxy-1,4-benzoxazin-3-one), the activity of P450s, GSTs, and CarEs (carboxylesterases) in fat bodies and midguts were significantly elevated [30]. Previous studies have shown that the midgut, fat body, and Malpighian tubules of silkworms fed with artificial diets exhibit elevated detoxification enzyme activities [9]. However, the expression patterns of related detoxification genes and the specific adaptation mechanisms of silkworms to the secondary metabolites in artificial diets have not been systematically elucidated.

In this study, we compared the adaptive phenotypic differences in silkworms fed with mulberry leaves and artificial diets. Through transcriptomic and metabolomic analyses, it was found that silkworms reared on artificial diets showed enrichment of detoxification functions in the fat body, midgut, and Malpighian tubules, as well as accumulation of secondary metabolites. Cellular level metabolite stimulation experiments revealed direct correlations between specific metabolites and detoxification gene expression. Using gene overexpression technology, the importance of the *GSTd2* gene in metabolic adaptation to flavonoids and artificial diets was verified at both cellular and individual levels. These findings can provide guidance for the optimization of artificial diet formulations and better artificial diet rearing practices of silkworms.

## 2. Materials and Methods

### 2.1. Experimental Insects and Diets

Individuals of the silkworm strain DaZao were provided by the Biological Science Research Center of Southwest University. The mulberry leaves were harvested from an experimental field at Southwest University (Chongqing, China). The artificial diet formula was the same as that used in a previous study: 30% mulberry leaf powder, 35% defatted soybean meal, 20% corn flour, 5% forming agent, 5% vitamins and inorganic salt complexes, and 5% other components [9]. Silkworm larvae reared on fresh mulberry leaves or artificial diets at 25 °C with 75 ± 5% relative humidity under a 12 h light/12 h dark photoperiod. At least 50 larvae were used per group in each independent experiment, and three independent biological replicates were performed.

### 2.2. RNA Extraction and Real-Time Quantitative PCR (RT-qPCR) Analysis

Total RNA extraction and quantitative RT-PCR analysis were performed as previously described [9]. Tissues were homogenized by electric homogenizer on ice and extracted using Trizol reagent (TRIzol™ Reagent, Invitrogen, Carlsbad, CA, USA). RT-qPCR was performed using SYBR qPCR SuperMix Plus (Novoprotein, Suzhou, China). Gene expression fold change was normalized against the expression of housekeeping gene *BmeIF4a* and the relative expression of the target gene was calculated using the 2^−ΔΔCt^ method. The sequences of the primers used in this study are provided in Table S1. Three independent biological replicates and three technical replicates were performed for each sample.

### 2.3. Setae Dispersion Rate Statistics

Use a digital camera to photograph the investigation area of silkworm larvae after feeding for 48 h. Open the images with drawing software, count the number of seta-dispersed silkworms and the total number of collected silkworms, and calculate according to the following formula: the percentage of setae dispersion (PST) at 48 h after feeding = (number of seta-dispersed silkworms/total number of collected silkworms) × 100%. The data from three independent experiments were analyzed and presented as the mean ± standard errors of the mean (*n* = 200).

### 2.4. Transcriptome and Metabolome Sequencing and Data Analysis

Library construction, transcriptome and metabolome sequencing were carried out by Shanghai Majorbio Bio-pharm Biotechnology Co., Ltd. (Shanghai, China), and data analysis was performed as previously described [9]. The transcriptome libraries were sequenced on an Illumina Novaseq 6000 platform (San Diego, CA, USA) and raw reads were filtered to obtain clean reads by removing adapters, poly-N and low-quality reads. The clean reads were mapped to the silkworm reference genome (https://kaikobase.dna.affrc.go.jp/KAIKObase_download.html, accessed on 12 June 2024). TPM (transcript-per-million) of each gene was calculated using the RSEM package (version 1.2.23) [31]. KEGG (Kyoto Encyclopedia of Genes and Genomes) pathway analysis of DEGs (different expression genes) was performed using KOBAS 3.0 software (http://kobas.cbi.pku.edu.cn/, accessed on 10 July 2024) [32]. The metabolome samples were analyzed using the ultra-high-performance liquid chromatography triple time-of-flight system from AB SCIEX. The separation was performed on an HSS T3 column (100 mm × 2.1 mm, 1.8 µm). The mobile phase consisted of solvent A (0.1% formic acid in water: acetonitrile) and solvent B (0.1% formic acid in acetonitrile: isopropanol: water). The gradient profile was as follows: 0–0.5 min (100% phase A), 0.5–2.5 min (0% to 25% phase B), 2.5–9 min (25% to 100% phase B), 9–13 min (100% phase B), 13–13.1 min (100% to 0% phase B), and 13.1–16 min (100% phase A). The column temperature was maintained at 40 °C. After mass spectrometry detection is completed, the raw data were preprocessed by Progenesis QI software (Waters Corporation, Milford, MA, USA). The metabolites were compared against the Human Metabolome Database, METLIN, and Majorbio database. Pathway analysis was performed using the KEGG database (http://www.genome.jp/kegg/, accessed on 15 April 2025). Enrichment analysis was performed using Python 2.7 scipy.stats package, and significantly enriched pathways were identified using *p* < 0.05. The original sequencing data generated in this study have been deposited in the National Center for Biotechnology Information Short Read Archive (accession number PRJNA923857 and MTBLS13782). For transcriptome and metabolome analysis, three and six independent biological replicates were performed for each group.

### 2.5. Prokaryotic Gene Expression and Protein Purification

To express the recombinant BmGSTd2 protein, a pair of specific primers (*BmGSTd2*-proF/R) containing the *Nhe*I and *BamH*I restriction sites at either end was used to amplify the *BmGSTd2* coding sequence (Supplemental Table S1). Subsequently, the PCR products and blank pET-28a vector (Novagen, Madison, WI, USA) were digested with the same enzymes *Nhe*I and *BamH*I, and the two digested products were ligated to construct the recombinant pET-28a-*GSTd2* plasmid. Next, the expression vectors pET-28a-*GSTd2* were transformed into the BL21 (DE3) competent cells. The recombinant colony was grown in LB medium and shaken at 37 °C until the optical density of the cells at 600 nm was between 0.4 and 0.6. Finally, the recombinant BmGSTd2 protein was induced by the addition of 0.1 mM isopropyl-β-d-thiogalactoside (IPTG) at 37 °C, 25 °C and 16 °C. After the bacteria were sonicated, the supernatant containing BmGSTd2 protein was then purified by nickel affinity column and eluted with imidazole. SDS-PAGE was used to analyze the expression and purity of the recombinant BmGSTd2 protein. Rabbit polyclonal antibody against BmGSTd2 was prepared by Zoonbio Biotechnology (Nanjing, China).

### 2.6. Protein Extraction and Western Blot Analysis

Proteins were extracted from transgenic and wild-type silkworms with RIPA lysis buffer (Beyotime, Shanghai, China) containing 1 mM PMSF on ice. Protein extracts were separated on a 10% SDS-PAGE gel and transferred onto polyvinylidene fluoride membranes (Roche, Indianapolis, IN, USA) using an electrotransfer apparatus (BioRad, Hercules, CA, USA). The membranes were blocked and then incubated with primary antibody (rabbit anti-BmGSTd2, dilution at 1/1000), protein blots were probed with goat Anti-rabbit IgG/HRP (Abcam, Shanghai, China) (dilution at 1/10,000). Protein bands were incubated with ECL detection kit (Thermo Fisher Scientific, Waltham, MA, USA) and visualized with the enhanced chemiluminescence system (NEN). α-tubulin served as a protein-loading control.

### 2.7. Cell Culture, Transfection and Viability Assay

The *B. mori* embryo cells (BmE) are widely used in the study of the silkworm [33,34]. BmE cells were cultured in Grace’s insect medium (Gibco, Gaithersburg, MD, USA) containing 10% fetal bovine serum (Gibco, Melbourne, Australia), 100 U/mL streptomycin, and 100 U/mL penicillin at 27 °C. The over-expression vector pSL1180-*GSTd2* was constructed to overexpress the silkworm *GSTd2* gene, which cotransfected into BmE cells using X-treme GENE HP DNA transfection reagent (Roche, Basel, Switzerland). Transfected cells were collected at 12 h, 18 h, and 24 h for gene expression analysis. To investigate the effects of different metabolites on cell viability, BmE cells of the silkworm were first seeded into 12-well plates by 5 × 10^5^ cells/well. Subsequently, the cells were treated with various concentrations of metabolites for 24–48 h, with DMSO-treated cells serving as the negative control. Cell viability was then assessed using the cell counting kit 8 (CCK-8) assay.

### 2.8. Obtaining Transgenic Silkworms

The *BmGSTd2* overexpression vector piggyBac [3 × P3-DsRed, IE2-*BmGSTd2*-SV40] were mixed with the same mass of pHA3PIG helper plasmid and subsequently injected into eggs of non-diapausing silkworm strain D9L via a microinjection system consisting of a micromanipulator (Eppendorf, Hamburg, Germany) and microinjection instrument (Eppendorf, Germany). The injected silkworm eggs (G_0_ generation) were sealed with instant glue and maintained at 25 °C and 90% RH. When the G_0_ generation silkworms had laid eggs and developed to the seventh day, an SZX16 fluorescence microscope (OLYMPUS, Tokyo, Japan) was used to screen the transgene-positive silkworms with red fluorescence in eyes.

### 2.9. Disk Diffusion Assay

A disk diffusion assay was carried out following Tang et al. [35]. A total of 200 μL of the *E. coli* BL21 (DE3)-CK and BL21 (DE3)-GSTd2 liquid medium (OD_600_ = 1) was distributed on LB (Luria–Bertani) agar plates (50 mg/L kanamycin, 1 mmol/L IPTG), and incubated at 37 °C for 1 h. Filter papers (5 mm diameter) were soaked in CHP solutions with 0, 50, 100, 200, and 300 mmol/L concentrations, and placed on the surface of LB agar plates, followed by incubation at 37 °C for 24–36 h. The inhibition zones were measured. Each concentration was repeated three times, and the experiment was conducted twice.

### 2.10. Developmental Parameters Measurement

The ratio of molting silkworm is the proportion of 3rd instar molting silkworms 12 days after feeding, which calculated using the formula: number of silkworms in the 3rd molting stage/total number of collected silkworms × 100%. Formula for calculating the uniformity of development of 4th/3rd instar larva: (Number of 4th/3rd instar molting silkworms/(Number of 4th/3rd instar molting silkworms + Number of non-molting silkworms + Number of 0 h of 5th/4th instar larva)) × 100%. Error bars represent the mean ± S.E.

### 2.11. Statistical Analysis

Data presentation was performed using GraphPad Prism version 9.0 (GraphPad Software, San Diego, CA, USA). The normality of the data was assessed by the Shapiro–Wilk test. Student’s *t* and chi-square test were applied to compare the significance between groups. Differences were considered statistically significant when *p* < 0.05.

## 3. Results

### 3.1. Impacts of Artificial Diet Rearing on Adaptive Phenotypes of Silkworms

**Figure 1:**
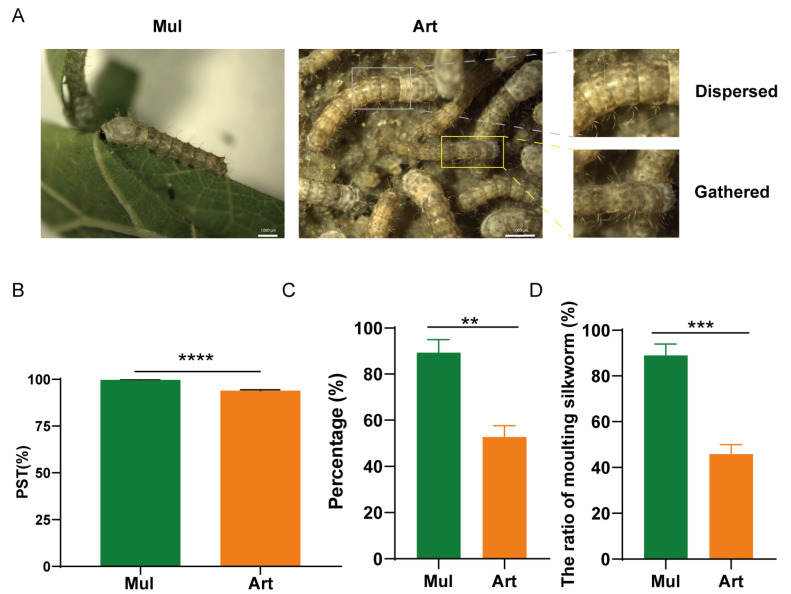
Comparison of setae dispersion rate and growth development between mulberry leaf-fed and artificial diet-fed silkworms. (**A**) Observation of silkworms after feeding for 48 h. (**B**) Statistics of setae dispersion rate. The body color of newly hatched silkworms is black or brown. When their body length reaches about 2–3 mm, their bodies are covered with setae. After feeding, their bodies enlarge, their color lightens, and their setae disperse. This process is called “setae dispersion”. PST: the percentage of setae dispersion. (**C**) Statistics on the uniformity of development in the 3rd instar fed with artificial diet. (**D**) The proportion of silkworms fed with artificial diet in the 3rd molting stage. The molting silkworms refers to silkworm larvae that has entered a non-feeding and immobile dormant state prior to ecdysis. Statistically significant differences are indicated as follows: ** *p* < 0.01, *** *p* < 0.001, **** *p* < 0.0001 (Chi-square test).

First, silkworms were fed with mulberry leaves and artificial diets for the whole life stage, and the setae dispersing condition of silkworms was observed 48 h after feeding. As shown in Figure 1A, almost all the body color of silkworms fed with mulberry leaves changed from black or brown to light color, while some silkworms fed with artificial diets still showed black, and the setae were not dispersed. The chi-square test indicated that the setae dispersion rate was 99.7% in silkworms reared on mulberry leaves, whereas it significantly decreased to 93.9% in those reared on artificial diets (Figure 1B). Furthermore, feeding and adaptability parameters of silkworms from the 1st to the 3rd instar were investigated. After 13 days feeding, the proportion of 3rd-molt silkworms in the artificial diet group was significantly decreased, and the 3rd instar developmental uniformity was also poor, which was reduced by 43.2% and 36.6% compared to mulberry leaf-reared silkworms (Figure 1C,D). These results suggest poor feeding and metabolic adaptability of silkworms to artificial diets.

### 3.2. Silkworms Show Distinct Transcriptomic and Metabolomic Alterations in Response to Diet Chang

**Figure 2:**
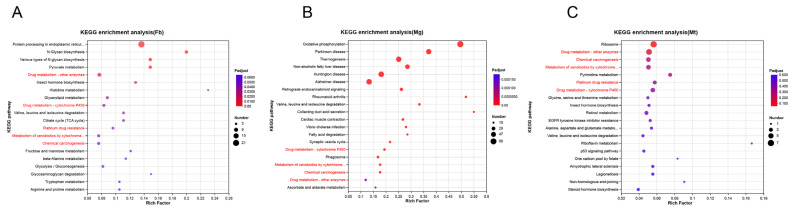
KEGG enrichment analysis scatterplot. Gene enrichment analysis of DEGs in Fb (**A**), Mg (**B**) and Mt (**C**). KEGG: Kyoto encyclopedia of genes and genomes. The red font in the figure indicates the detoxification metabolism pathway. Fb: fat body; Mg: midgut; Mt: Malpighian tubule.

Transcriptomic and metabolomic analyses were further performed on the fat body, midgut, and Malpighian tubules of silkworms reared under different dietary conditions. Transcriptome KEGG enrichment analysis of significantly upregulated genes in artificial diet-fed silkworms revealed the enrichment of multiple detoxification pathways across all three tissues, including drug metabolism-other enzymes, drug metabolism-cytochrome P450, metabolism of xenobiotics by cytochrome P450, chemical carcinogenesis, and platinum drug resistance (red font, Figure 2). Numerous detoxification genes were upregulated in these pathways, particularly members of the *CYP*, *GST*, *UGT*, and esterase families (Supplementary Table S2). Specifically, 13 genes were upregulated in the fat body (*CYP18a/4L/6A/6B*, *GSTd2*, *microGST* and *UGT40G/40H*), 13 in the midgut (*CYP9006/4678*, *GSTd2/e*, *microGST* and *UGT40B/41A*), and 23 in Malpighian tubule (*CYP333un/6A/49A/4S5/340C*, *GSTd2/e/o*, *UGT33D/40K/40G/42A/46A*).

In metabolomic analysis, using VIP > 1, FC > 1, and *p* < 0.05 as screening criteria, multiple secondary metabolites were identified as significantly upregulated in the midgut, Malpighian tubule, and fat body. These included one benzoate compound; three flavonoids, including isoflavone and flavonoid glycoside; three saponins; seven terpenoids, including monoterpenoid, triterpenoid, sesterterpenoid, and terpene glycoside; and two lipid compounds, including steroid lactone and steroidal glycoside. Among them, the benzoates substance propyl gallate showed the highest upregulation (564.9-fold), followed by 3beta-3-Hydroxy-18-lupen-21-one (104.2-fold) and glycitin (8.9-fold). In addition, the flavonoid glycoside pelargonidin 3-*O*-glucoside and flavonoid apigetrin were upregulated by 2.3-fold and 2-fold, respectively (Table 1).

### 3.3. Changes in Detoxification Genes of Silkworm BmE Cells in Response to Metabolite Stimulation

To investigate the potential association between significantly upregulated metabolites and detoxification genes in omics, nine metabolites from four categories (six flavonoids, one saponin, one flavonoid glycoside, and one benzoate) were used to stimulate silkworm BmE cells. RT-qPCR analysis was subsequently performed on *GST*, *CYP*, and *UGT* detoxification genes that were upregulated in the transcriptome.

**Figure 3:**
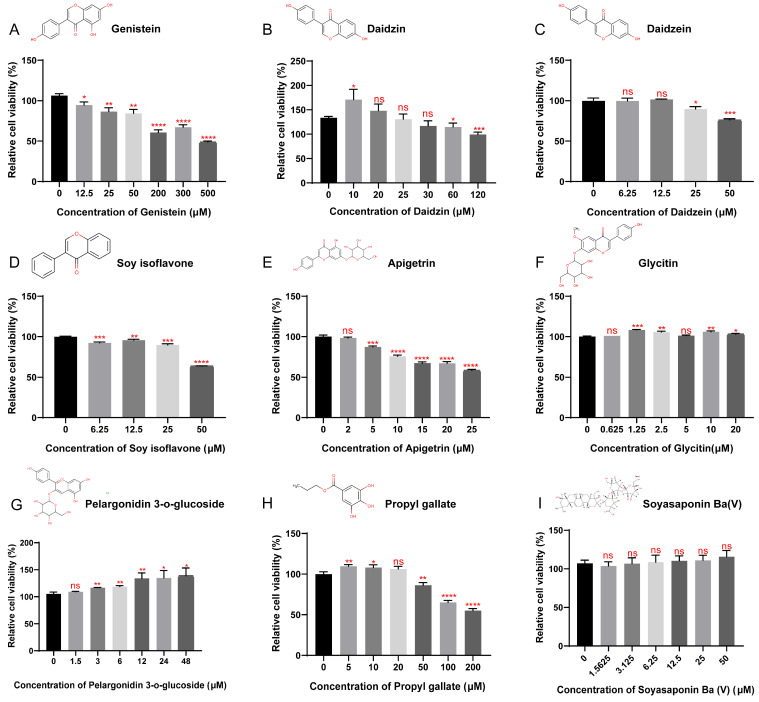
Detection of relative cell viability after treatment with six potential metabolic stimuli. The structural diagram in the figure is a chemical structural description of six potential metabolic stimuli, which are Genistein (**A**), Daidzin (**B**), Daidzein (**C**), Soy isoflavone (**D**), Apigetrin (**E**), Glycitin (**F**), Pelargonidin 3-*O*-glucoside (**G**), Propyl gallate (**H**) and Soyasaponin Ba (V) (**I**). Statistically significant differences are indicated as follows: * *p* < 0.5, ** *p* < 0.01, *** *p* < 0.001, **** *p* < 0.0001, ns: no significant difference (Student’s *t*-test).

Cell viability assays showed that among the flavonoids, genistein (Figure 3A), daidzin (Figure 3B), daidzein (Figure 3C), soy isoflavone (Figure 3D) and apigetrin (Figure 3E) inhibited BmE cell viability at concentrations of 12.5, 60, 25, 6.25, and 5 μM, respectively. Propyl gallate exhibited cell inhibitory activity at 50 μM (Figure 3H). In contrast, glycitin (Figure 3F) and pelargonidin-3-*O*-glucoside (Figure 3G) enhanced cell viability at 1.25 and 3 μM, respectively, while soyasaponin Ba (V) (Figure 3I) had no significant effect on cell viability.

**Figure 4:**
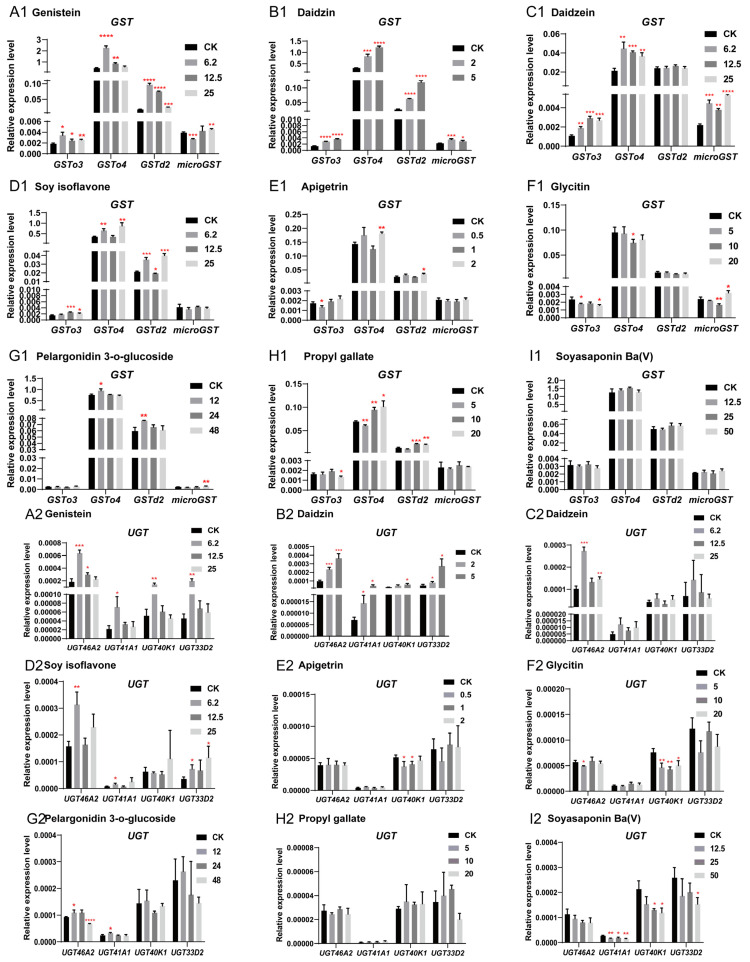

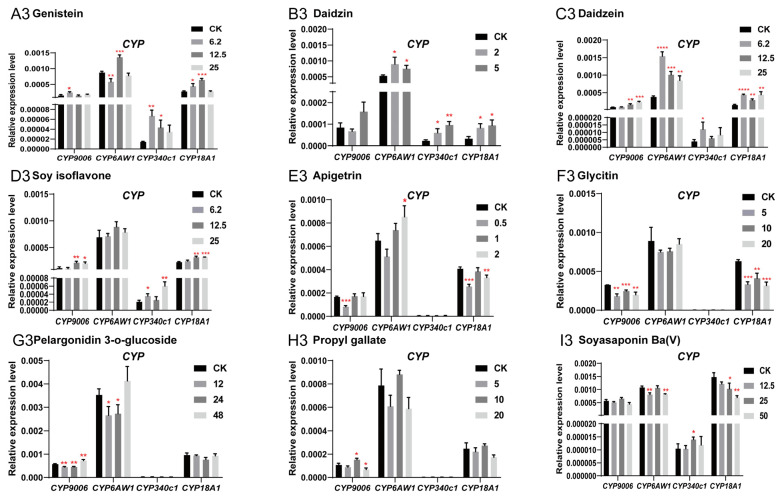
Analysis of the expression patterns of detoxification genes *GST*, *UGT*, and *CYP* in BmE cells induced by six metabolic stimuli. (**A1–3**) Expression patterns of *GST*, *UGT*, and *CYP* detoxification genes in BmE cells induced by stimulation with genistein. (**B1–3**) Expression patterns of GST, *UGT*, and *CYP* detoxification genes in BmE cells induced by stimulation with daidzin. (**C1–3**) Expression patterns of *GST*, *UGT*, and *CYP* detoxification genes in BmE cells induced by stimulation with daidzein. (**D1–3**) Expression patterns of *GST*, *UGT*, and *CYP* detoxification genes in BmE cells induced by stimulation with soy isoflavone. (**E1–3**) Expression patterns of *GST*, *UGT*, and *CYP* detoxification genes in BmE cells induced by stimulation with apigetrin. (**F1–3**) Expression patterns of *GST*, *UGT*, and *CYP* detoxification genes in BmE cells induced by stimulation with glycitin. (**G1–3**) Expression patterns of *GST*, *UGT*, and *CYP* detoxification genes in BmE cells induced by stimulation with pelargonidin 3-*O*-glucoside. (**H1–3**) Expression patterns of *GST*, *UGT*, and *CYP* detoxification genes in BmE cells induced by stimulation with propyl gallate. (**I1–3**) Expression patterns of *GST*, *UGT*, and *CYP* detoxification genes in BmE cells induced by stimulation with soyasaponin Ba (V). In the figure, the black CK bars represent the expression levels of detoxification genes in untreated cells, while the gray bars of varying shades represent the expression levels of detoxification genes in cells treated with different concentrations of the drug. The unit of drug concentration is μM. Statistically significant differences are indicated as follows: * *p* < 0.5, ** *p* < 0.01, *** *p* < 0.001, **** *p* < 0.0001 (Student’s *t*-test).

The results of RT-qPCR detection are shown in Figure 4, with a summary of detoxification gene responses in BmE cells under different metabolite stimulation presented in Supplementary Table S3. In the table, red, green, and black fonts denote gene upregulation, downregulation, and no significant change in response to the test substances, respectively. As shown in the table, among flavonoids, genistein, daidzin, soy isoflavone, and daidzein exhibited obvious inductive effects on cells, inducing the upregulated expression of 12, 11, 9, and 8 detoxification genes, respectively. Treatment with the flavonoid glycoside pelargonidin-3-*O*-glucoside, benzoate propyl gallate, flavonoids apigetrin and glycitin, and saponin soyasaponin Ba (V) induced upregulation of 5, 3, 3, 1, and 1 genes, respectively. Analysis of major responsive gene families revealed that *GSTo4* and *GSTd2* were the most frequently induced, showing upregulation in 7 and 6 times, respectively. Moderate responsiveness was observed for *microGST*, *CYP340C1*, and *UGT46A2*, each upregulated 5 times. *GSTo3*, *CYP9006/6AW1/18A1*, *UGT33D2*, and *UGT41A1* were upregulated 4 times, while *UGT33D2* and *UGT40K1* exhibited low-level induction, and were upregulated 3 and 2 times, respectively. Collectively, these results support a function association between significantly upregulated metabolites and detoxification genes in the omics.

### 3.4. GSTd2 Was Significantly Upregulated by Induction of Artificial Diet Rearing

**Figure 5:**
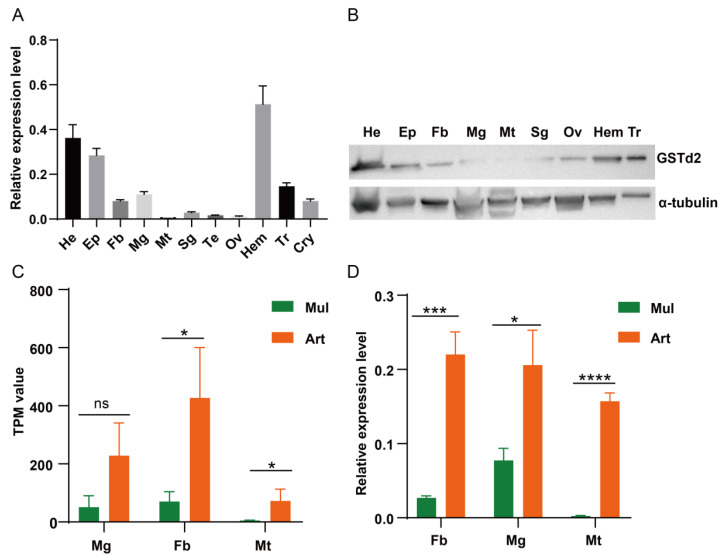
Expression profile analysis of *GSTd2* in silkworms reared on mulberry leaves and artificial diet. The tissue expression profile (**A**) and protein expression profile. (**B**) of the *GSTd2* in silkworms fed with mulberry leaves. (**C**) Expression level (TPM) of *GSTd2* identified in the transcriptome of silkworms fed with mulberry leaves and artificial diet. (**D**) Comparison of *GSTd2* expression in silkworms fed with mulberry leaves and artificial diet. He: head; EP: epidermis; Fb: fat body; Mg: midgut; Mt: Malpighian tubules; Sg: silk gland; Te: testis; Ov: ovary; Hem: hemocyte; Tr: trachea; Cyr: cryptonephry. Statistically significant differences are indicated as follows: * *p* < 0.5, *** *p* < 0.001, **** *p* < 0.0001, ns: no significant difference (Student’s *t*-test).

In the detoxification metabolic pathways enriched by transcriptome KEGG analysis, we found that only one detoxification gene, *GSTd2*, was upregulated in the fat body, midgut, and Malpighian tubules. RT-qPCR and Western blot analyses revealed that under mulberry leaf feeding, *GSTd2* gene was mainly expressed in the head, epidermis, and hemocyte tissues, with lower expression levels in detoxification tissues such as the fat body, midgut, and Malpighian tubules (Figure 5A,B). By contrast, under artificial diet feeding, the expression level of *GSTd2* gene was significantly upregulated in response to dietary changes, and the RT-qPCR results were consistent with the transcriptome data (Figure 5C,D).

To better study the function of the *GSTd2* gene, the full-length coding sequence of *GSTd2* was cloned into the PET28a expression vector for prokaryotic expression (Figure S1A,B). The recombinant protein was expressed in both the supernatant and the precipitate at all three induction temperatures (16, 25, and 37 °C), with the highest expression level observed in the supernatant at 25 °C (Figure S1C). The recombinant protein was purified by nickel column affinity chromatography and eluted at imidazole concentrations of 100, 200 and 300 mM (Figure S1D). Subsequently, the purified recombinant protein was sent to Zoonbio Biotechnology Company for the development of polyclonal antibody in rabbit.

### 3.5. Silkworm BmE Cells Overexpressing GSTd2 Exhibit Enhanced Metabolic Adaptation to Flavonoids

**Figure 6:**
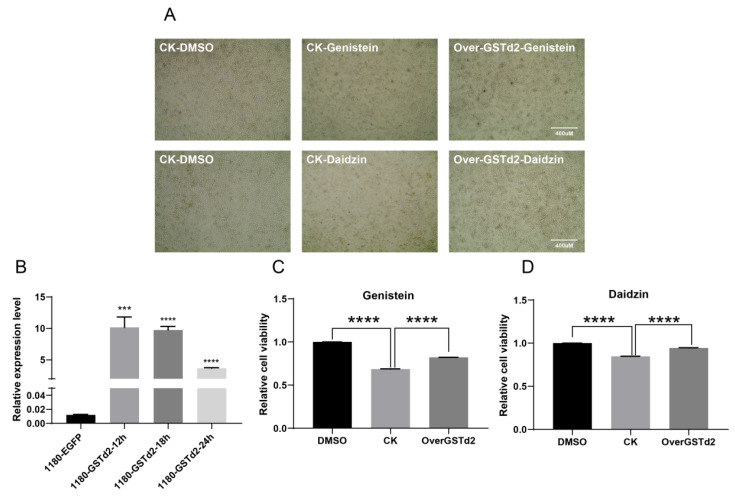
Response of BmE cells overexpressing *GSTd2* gene to genistein and daidzin. (**A**) Comparative observation of *GSTd2* overexpression and WT cells after treatment with genistein and daidzin. (**B**) Detection of *GSTd2* expression level at different time points in BmE cells. (**C**) The relative viability of cells after treatment with genistein. (**D**) The relative viability of cells after treatment with daidzin. Statistically significant differences are indicated as follows: *** *p* < 0.001, **** *p* < 0.0001 (Student’s *t*-test).

In order to analyze whether changes in the *BmGSTd2* gene affect the response of BmE cells to metabolic stimuli, we overexpressed *BmGSTd2* at the cellular level and observed the cells morphology (Figure 6A). RT-qPCR analysis of *BmGSTd2* expression revealed significant overexpression at 12, 18, and 24 h post-transfection, with peak induction observed at 12 h (Figure 6B). Based on the results of metabolic stimulation experiment, genistein and daidzin showed the strongest induce of the *GSTd2* gene in the quantitative results, which were selected as metabolic stimulants to treat both *BmGSTd2*-overexpressing and wild-type cells, with DMSO serving as the negative control. The cell viability assay results showed that BmE cells overexpressing *BmGSTd2* exhibited stronger cell viability and tolerance compared to control cells when challenged with either genistein or daidzin (Figure 6C,D).

### 3.6. Overexpression of GSTd2 Gene in Silkworms

**Figure 7:**
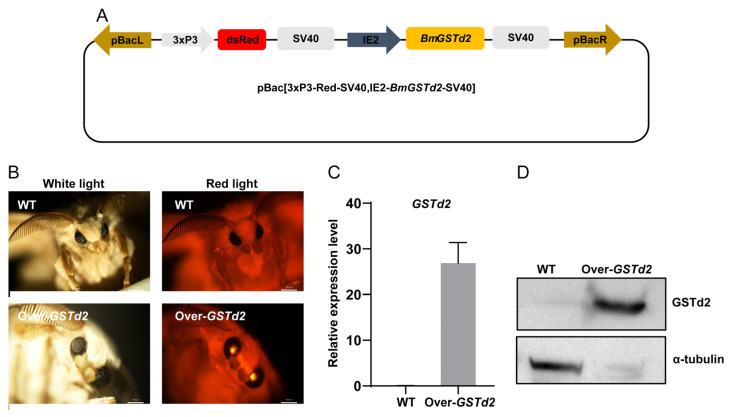
Vector schematic diagram and screening and detection of positive individuals. (**A**) Schematic diagram of carrier skeleton. (**B**) Screening for positive individuals. (**C**) Transcription level detection of *GSTd2*. (**D**) Protein level detection of GSTd2 in WT and Over-*GSTd2* silkworms. WT: wild-type silkworms; Over-*GSTd2*: *BmGSTd2* overexpression silkworms.

After discovering that overexpression of the *GSTd2* gene at the cellular level could confer stronger metabolic adaptability to genistein and daidzin in BmE cells, we extended overexpressing *GSTd2* gene at the individual level to further investigate whether changes in the *GSTd2* gene at the individual level would affect the metabolic adaptability of silkworms to artificial diets. The piggyBac-mediated germline transformation was used to overexpress *BmGSTd2* in D9L silkworms. The piggyBac transgenic vector was constructed, containing pBac[IE2-*BmGSTd2*-SV40] and pBac[3xP3-Red-SV40] (Figure 7A). The transgenic vector was co-injected with the piggyBac helper plasmid into silkworm eggs, and individuals with red fluorescence eyes were screened from their offspring (Figure 7B). Furthermore, RT-qPCR and Western blot analyses were used to detect the mRNA and protein levels of *GSTd2* gene, and it was found that *GSTd2* gene was significantly overexpressed in the transgenic silkworms (Figure 7C,D).

### 3.7. Transgenic Overexpression of GSTd2 Enhances the Metabolic Adaptation of Silkworms to Flavonoids and Artificial Diets

**Figure 8:**
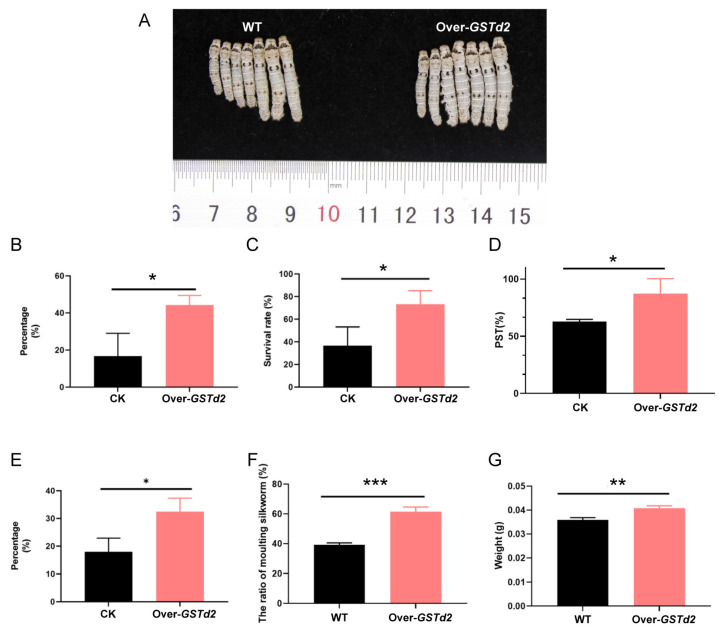
Statistics on the growth and development of WT and Over-*GSTd2* silkworms fed with artificial diet and a diet supplemented with flavonoids. (**A**) Phenotype observation of newly molted fifth instar larvae fed with artificial diet. (**B**) The proportion of newly molted second instar larvae after feeding for 96 h for newly hatched fed with artificial diet supplemented with flavonoids. (**C**) Survival rate of silkworms fed with artificial diet supplemented with flavonoids for two weeks. (**D**) Statistics on the percentage of setae dispersion (PST) after feeding for 48 h for newly hatched larvae fed with artificial diet. (**E**) Statistics on the uniformity of development in the 4th instar fed with artificial diet. (**F**) The proportion of silkworms fed with artificial diet in the 3rd molting stage. (**G**) The weight of silkworms fed with artificial diet on the 3rd molting stage. WT: wild-type silkworms; Over-GSTd2: BmGSTd2 overexpression silkworms. Statistically significant differences are indicated as follows: * *p* < 0.5, ** *p* < 0.01, *** *p* < 0.001 (Chi-square test).

Following successful overexpression of the *GSTd2* gene, the tolerance of silkworms to flavonoids was first examined. Wild-type and overexpressing silkworms were reared on an artificial diet containing 6250 ppm of soy isoflavones. The results showed that the proportion of silkworms rising after molting in the group with *GSTd2* gene overexpression was 20% higher than that in the wild-type group after feeding for 96 h for newly hatched (Figure 8B), and the survival rate after two weeks was also 40% higher than that in the wild-type group (Figure 8C).

Subsequently, the adaptability of wild-type and Over-*GSTd2* silkworms to artificial diet was investigated. Phenotypic observations revealed that the epidermal color of Over-*GSTd2* newly molted fifth instar larvae reared on artificial diet was whiter than that of wild-type silkworms, whose epidermis exhibited an unhealthy light yellowish in color (Figure 8A). The results showed that the PST in the *GSTd2*-overexpression group was 38.6% higher than that in the wild-type group after feeding for 48 h for newly hatched (Figure 8D). When the silkworms reach the 4th instar, the developmental uniformity in the overexpression line exhibited an 16% increase compared to wild-type silkworms (Figure 8E). At 12 days after hatching, the proportion of silkworms in the 3rd molting stage was analyzed as a parameter reflecting developmental speed, and it was found that Over-*GSTd2* silkworms were 23.3% higher than the wild-type silkworms (Figure 8F). Additionally, Over-*GSTd2* silkworms showed a 13.5% increase in mean body weight compared to wild-type individuals in the 3rd molting stage (Figure 8G).

## 4. Discussion

Generally, insects respond to exogenous substances by directly avoiding them through olfactory or visual means [36,37], or by excreting or sequestering the compounds to prevent harm [38,39]. However, detoxification metabolism of xenobiotics remains the dominant adaptive mechanism for insects to utilize novel food sources [2,3]. In this study, we found that a large number of detoxification genes were up-regulated in the midgut, fat body and Malpighian tubules of silkworms reared on artificial diets, including *CYP*, carboxylesterase, *GST* and *UGT*. This likely represents a metabolic adaptation mechanism for silkworms to counteract potential toxins in artificial diets. It is reported that in the proteome study of the midgut of silkworms reared on artificial diets and mulberry leaves by Zhang et al., key detoxification-related differential proteins UGT40B4, UGT340C2, and UGT40A1 were identified [40]. Among them, *UGT40B4* was also detected in the midgut transcriptome of this study. Additionally, a genomic study by Xin et al. on the artificial diet-adapted silkworm strain Guican No.5 identified new genes contributing to silkworm adaptation to artificial diets, including *CYP450*, carboxylesterase, heat shock protein (MSTRG.2920), and copper/zinc superoxide dismutase (MSTRG.1993) [41]. These findings not only corroborate the involvement of detoxification genes in metabolic adaptation mechanisms, but also indicate that in addition to this classic adaptive evolution model, there exist different adaptive strategies related to oxidative stress and antioxidant responses. It is worth mentioning that in the supplementary study on the function of *GSTd2*, strain *GSTd2* over-expression exhibited a higher survival rate under stress of the organic peroxide CHP (cumene hydroperoxide), with the size of the inhibition zone significantly smaller than that of the control group (Supplementary Figure S2). This suggests that the silkworm *GSTd2* also possesses peroxidase activity and may be involved in the metabolism of peroxidation products in artificially fed silkworms.

In artificial diets, soybean powder and cornmeal not only contain nutrients such as proteins and carbohydrates but also include various secondary metabolites, such as soybean isoflavones genistein, daidzein, and daidzin [42,43]. Studies have shown that genistein present in the diet can regulate the expression and catalytic activity of *GSTs* in human breast cells [44]. Study by Fei et al. also demonstrated that genistein transcriptionally activates the rat glutathione S-transferase Ya (*rGSTYa*) subunit gene in a time- and dose-dependent manner [45]. Although insect *GST* gene families differ from those in humans, rats, and other mammals, the above results still proves to some extent that *GST* genes are involved in the metabolism of flavonoids such as genistein. In fact, the induction of detoxification genes by secondary metabolites like flavonoids and terpenoids is well-documented. For example, Luque et al. reported that *Bombyx mori UGT* has a broad substrate specificity for flavonoids, coumarins, terpenoids and simple phenols [46]. Allelochemicals such as terpenoids, flavonoids, and alkaloids have been confirmed to also promote the expression of *CYP* and *GST* genes [47,48,49]. Studies on the green peach aphid (*Myzus persicae*) have demonstrated that GST promotes the metabolism of glucosinolates and isothiocyanates, which are abundant in cruciferous plants [50]. Similar to the above results, the upregulation of detoxification genes in the silkworm is strongly related to the secondary metabolites such as flavonoids enriched in artificial diets. Future efforts to improve artificial diet efficiency for silkworm rearing may prioritize feed protein sources with reduced flavonoid and other allelochemical content. In addition, the presence of propyl gallate in artificial diet also has a stimulating effect on silkworms. Stimulation experiments on BmE cells showed that this substance mainly causes the up-regulated expression of *GST* genes (Figure 4). Propyl gallate is a synthetic preservative added to artificial diet to protect such oil- or fat-containing foods from oxidative rancidity [51]. Studies have shown that the addition of synthetic preservatives such as ethylparaben (EP, a chemical preservative) to artificial diet also induces upregulation of the detoxification gene *Ugt2* and the immune gene *Cecropin B*. In contrast, the addition of natural preservatives has no negative impact on the productivity of silkworms and the homeostasis of their gut microbiota [52]. Furthermore, studies by Zhao et al. indicated that propyl gallate detected in the midgut, hemolymph, and silk glands of silkworms reared on artificial diet has a negative effect on silkworm silk production [53]. Therefore, choosing preservatives that silkworms are more tolerant to, such as natural preservatives, may be a more suitable choice.

GSTs are critical phase II detoxification enzyme in organisms, primarily functioning to metabolize xenobiotics or endogenous toxic substances, thereby achieving detoxification [54,55]. In insects, beyond these canonical functions, GSTs also mediate insecticide resistance, olfactory response, and oxidative stress responses [56,57,58,59]. Although this study has found that *GSTd2* is crucial for the adaptation of silkworms to artificial diets, several limitations warrant mention. First, in addition to *GSTd2*, other detoxification genes identified in transcriptomics and BmE cells metabolite stimulation experiments may also contribute to metabolic adaptation to artificial diets, such as *GSTo4*, *microGST*, *CYP340C1*, and *UGT46A2*. For these key differential genes, further gene knockout or overexpression experiments are also required to establish their causal relationship with artificial diet adaptation. Second, the literature reports show that the mechanism of action of GST is to catalyze the conjugation of electrophilic compounds to the thiol group of GSH (reduced glutathione), thereby making the generated products more water-soluble than substances without GSH binding and excreting them from the body [59,60]. Additionally, some GSTs can utilize GSH as a cofactor to catalyze dehydrochlorination reactions for detoxification purposes [61]. In this study, overexpression of *GSTd2* may enhance such reactions in silkworms, thereby improving their adaptability to artificial diets. However, the underlying metabolic adaptation mechanism still requires further functional verification.

## 5. Conclusions

Focusing on larvae of the silkworm (*Bombyx mori*), using transcriptomic and metabolomic sequencing combined with differential analysis, we found that the detoxification functions in the fat body, midgut, and Malpighian tubules were significantly activated in silkworms reared on artificial diet, with a large number of *GST*, *UGT*, and *CYP* genes upregulated. Meanwhile, various secondary metabolites, such as flavonoids and terpenoids, were enriched in the tissues of silkworms reared on artificial diet. Further stimulation experiments in BmE cells revealed a correlation between the upregulated metabolites and detoxification genes. Among these metabolites, flavonoids including genistein and daidzin exerted the most significant stimulatory effects on cells, inducing the upregulation of various *GST*, *UGT*, and *CYP* genes. Functional assays confirmed the critical role of *GSTd2*: overexpression of *GSTd2* at the cellular level enhanced the tolerance of BmE cells to the flavonoids genistein and daidzin. Silkworms with systemic overexpression of *GSTd2* were obtained, and rearing experiments on artificial diet demonstrated that overexpression of this gene significantly improved the adaptability of silkworms to artificial diet. These findings provide novel insights into the metabolic adaptation of silkworms to artificial diet. Future research should focus on optimizing diet formulation and breeding silkworm varieties with improved diet adaptability, translating these molecular insights into practical solutions for enhancing silkworm rearing efficiency.

## Figures and Tables

**Table 1:** Metabolites significantly up-regulated in the midgut, Malpighian tubules and fat body of silkworms fed with artificial diets.

		Mg			Mt			Fb		
Class	Metabolite	VIP	FC (A/M)	*p*-Value	VIP	FC (A/M)	*p*-Value	VIP	FC (A/M)	*p*-Value
Benzoate	Propyl gallate	ND	ND	ND	2.8115	**564.901**	2.64 × 10^−14^	ND	ND	ND
Isoflavone	Glycitin	2.9445	**8.9807**	1.88 × 10^−26^	0.9527	1.1639	1.96 × 10^−6^	ND	ND	ND
Flavonoid	Apigetrin	ND	ND	ND	2.3895	**2.0253**	4.76 × 10^−13^	ND	ND	ND
flavonoid glycoside	Pelargonidin 3-*O*-glucoside	2.408	1.8684	2.28 × 10^−13^	1.8203	**2.3313**	1.38 × 10^−7^	ND	ND	ND
Saponin	Soyasaponin II	2.4618	1.9964	7.08 × 10^−13^	ND	ND	ND	ND	ND	ND
Saponin	Soyasaponin A2	2.0574	1.7053	3.01 × 10^−11^	ND	ND	ND	ND	ND	ND
Saponin	Soyasaponin Ba (V)	1.6687	1.3514	7.15 × 10^−11^	ND	ND	ND	ND	ND	ND
Monoterpenoid	Caryoptosidic acid	2.0506	1.9241	7.23 × 10^−17^	1.0199	0.8274	0.0002	ND	ND	ND
Terpene glycoside	Pitheduloside D	1.8418	1.7038	2.88 × 10^−5^	ND	ND	ND	ND	ND	ND
Terpene glycoside	Neryl rhamnosyl-glucoside	1.3349	1.2098	9.81 × 10^−15^	ND	ND	ND	ND	ND	ND
Triterpenoid	3beta-3-Hydroxy-18-lupen-21-one	1.6279	1.3485	1.06 × 10^−5^	2.7671	**104.297**	1.17 × 10^−10^	ND	ND	ND
Triterpenoid	Lucidenic acid M	1.8698	1.5409	2.26 × 10^−19^	ND	ND	ND	ND	ND	ND
Triterpenoid	Fasciculic acid A	2.0666	1.7744	3.17 × 10^−21^	ND	ND	ND	ND	ND	ND
Steroid lactone	Bipindogulomethyloside	1.6077	1.3214	2.64 × 10^−11^	ND	ND	ND	ND	ND	ND
Steroidal glycoside	Cortolone-3-glucuronide	1.9374	1.6306	1.60 × 10^−9^	ND	ND	ND	ND	ND	ND
Sesterterpenoid	25-Acetylvulgaroside	ND	ND	ND	ND	ND	ND	1.3222	1.0784	6.07 × 10^−5^

Text in bold in the table indicates a fold change greater than 2. ND: not detected.

## Data Availability

The original contributions presented in this study are included in the article. Further inquiries can be directed to the corresponding author.

## Supplementary Materials

The following supporting information can be downloaded at https://www.mdpi.com/article/10.3390/insects17030261/s1, Figure S1: Preparation and purification of GSTd2 recombinant protein. Figure S2: Disk diffusion assays using *E. coli* overexpressing BmGSTd2. Figure S3: The original image of the Western blot. Table S1: Primers used in the study. Table S2: Detoxification genes enriched in KEGG pathways in the fat body, midgut, and Malpighian tubules. Table S3: Relationship between xenobiotic metabolites and detoxification genes at the cellular level. Table S4: List of up-regulated genes in the fat body of silkworms fed with artificial diets compared to those fed with mulberry leaves in the transcriptome analyses. Table S5: List of up-regulated genes in the midgut of silkworms fed with artificial diets compared to those fed with mulberry leaves in the transcriptome analyses. Table S6: List of differential metabolites identified in the metabolome analyses of fat body. Table S7: List of differential metabolites identified in the metabolome analyses of midgut.
