# Supplementary material for: Detoxification Metabolic Adaptation of Bombyx mori to Artificial Diet and Functional Study of Key Detoxification Gene BmGSTd2
Source: Insects. 2026 Feb 28;17(3):261. doi: 10.3390/insects17030261 (PMC13027093; doi:10.3390/insects17030261)
Supplement: Supplementary file 1 [file insects-17-00261-s001.zip › Figure S1.pdf]

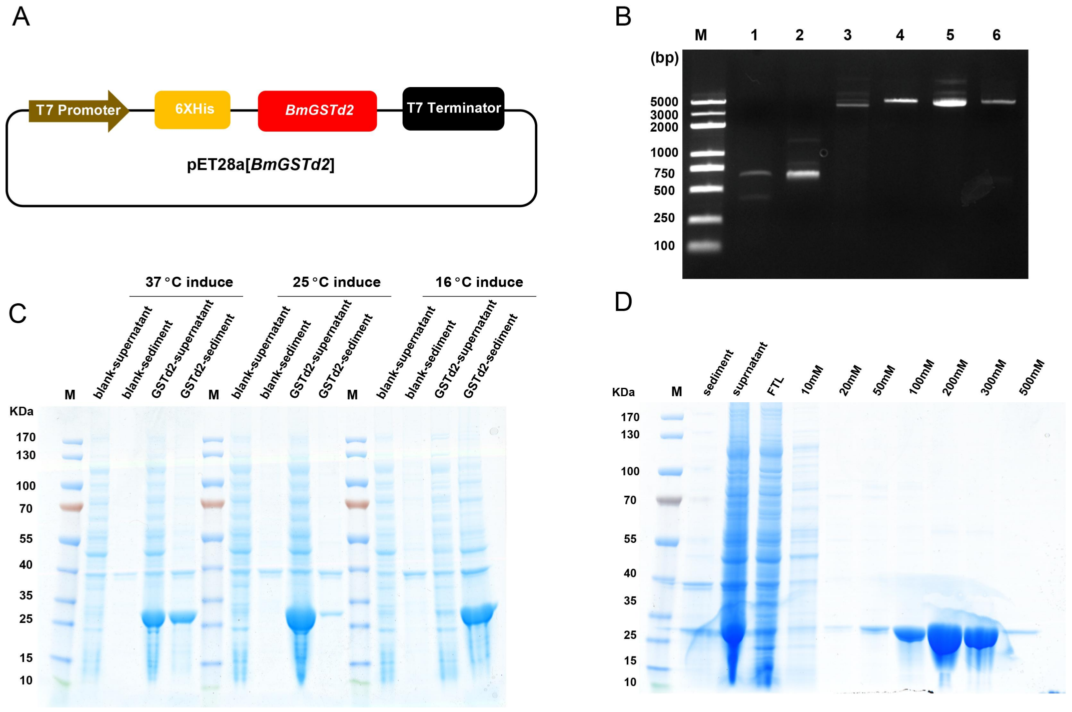

Figure S1. Preparation and purification of GSTd2 recombinant protein. (A) Construction of the prokaryotic expression vector pET28[BmGSTd2]. (B) Amplification and enzyme digestion verification of GSTd2. M: Marker; 1: The amplification product of *GSTd2* gene; 2: The amplification product of *GSTd2* gene digested with *Nhe* I and *BamH* I; 3: pET-28a vector; 4: pET-28a vector digested by *Nhe* I and *BamH* I; 5: pET-28a-BmGSTd2 vector; 6: pET-28a-BmGSTd2 vector digested by *Nhe* I and *BamH* I. (C) Prokaryotic expression of GSTd2 induced at different temperatures. (D) Purification of GSTd2 recombinant protein with different imidazole concentrations.
