# Supplementary material for: Detoxification Metabolic Adaptation of Bombyx mori to Artificial Diet and Functional Study of Key Detoxification Gene BmGSTd2
Source: Insects. 2026 Feb 28;17(3):261. doi: 10.3390/insects17030261 (PMC13027093; doi:10.3390/insects17030261)
Supplement: Supplementary file 1 [file insects-17-00261-s001.zip › Figure S2.pdf]

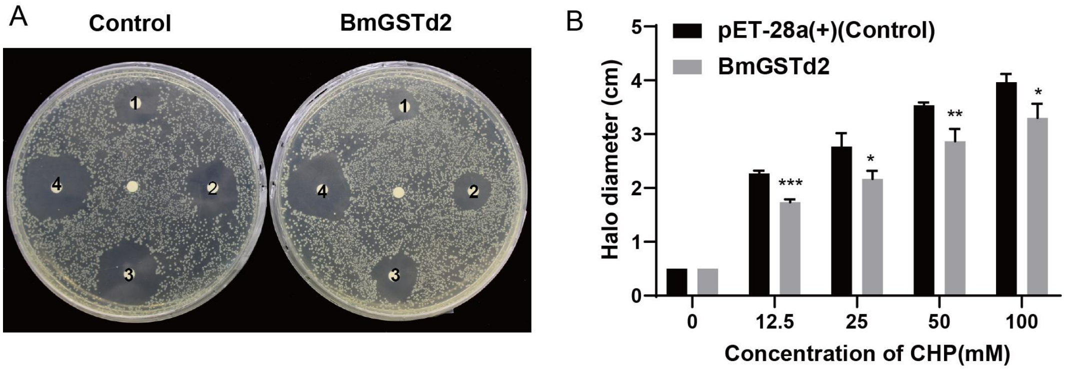


**Figure S2.** Disc diffusion assays using E. coli overexpressing BmGSTd2. (A) Inhibition zone halo diameter of *E.coli* expressing pET-28a-BmGSTd2 and pET-28a under different concentration of CHP. (B) Histograms comparing halo diameters of inhibition zones. Labels 0-4: 0, 12.5, 25, 50, 100 mmol/L CHP. Statistically significant differences are indicated as follows: *$p<0.5$, **$p<0.01$, ***$p<0.001$ (Student's *t*-test).
