# Supplementary material for: Detoxification Metabolic Adaptation of Bombyx mori to Artificial Diet and Functional Study of Key Detoxification Gene BmGSTd2
Source: Insects. 2026 Feb 28;17(3):261. doi: 10.3390/insects17030261 (PMC13027093; doi:10.3390/insects17030261)
Supplement: Supplementary file 1 [file insects-17-00261-s001.zip › Figure S3-The original image of the Western blot.pdf]

Figure 5B- GSTd2 gene

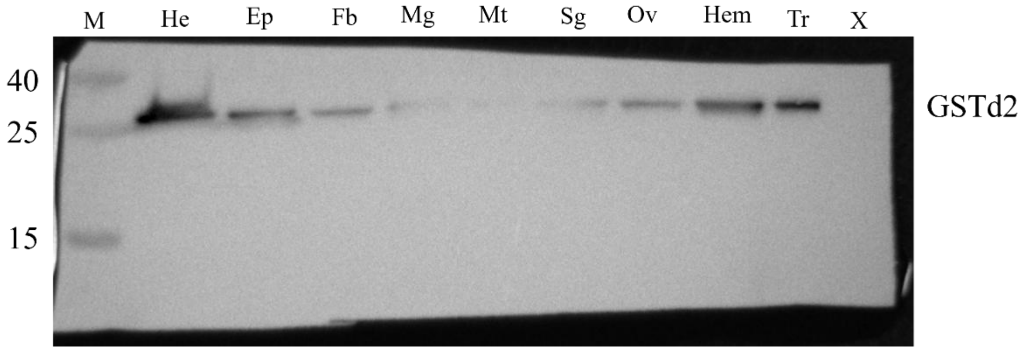

Protein expression profile analysis of the *GSTd2* in various tissues of the silkworm fed with mulberry leaves. He: head; EP: epidermis; Fb: fat body; Mg: midgut; Mt: Malpighian tubules; Sg: silk gland; Ov: ovary; Hem: hemocyte; Tr: trachea. X lane: the lane not used in the article.

Figure 5B-tubulin gene

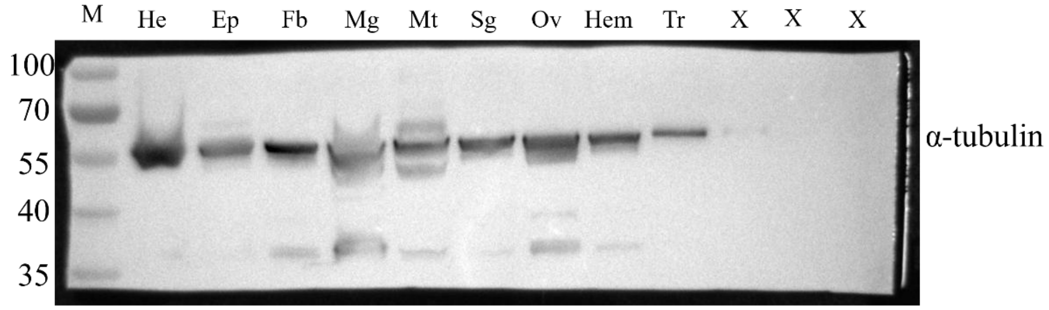

Protein expression profile analysis of α-tubulin in various tissues of the silkworm fed with mulberry leaves. He: head; EP: epidermis; Fb: fat body; Mg: midgut; Mt: Malpighian tubules; Sg: silk gland; Ov: ovary; Hem: hemocyte; Tr: trachea. X lane: the lane not used in the article.

Sample	GSTd2(mean)	α-tubulin(mean)	Ratio (GSTd2/α-tubulin)
He	48.656	54.399	0.894428206
Ep	18.190	34.374	0.529179031
Fb	7.638	51.924	0.147099607
Mg	2.737	24.403	0.112158341
Mt	1.302	30.220	0.04308405
Sg	3.626	45.923	0.078958256
Ov	7.789	51.598	0.150955463
Hem	21.531	39.818	0.540735346
Tr	16.263	23.031	0.706135209

Quantitative Immunoblot Analysis of GSTd2 and α-tubulin in silkworms. GSTd2(mean): densitometric measurement of GSTd2 protein band intensity. α-tubulin(mean): densitometric measurement of α-tubulin protein band intensity.

Figure 7D- GSTd2 gene

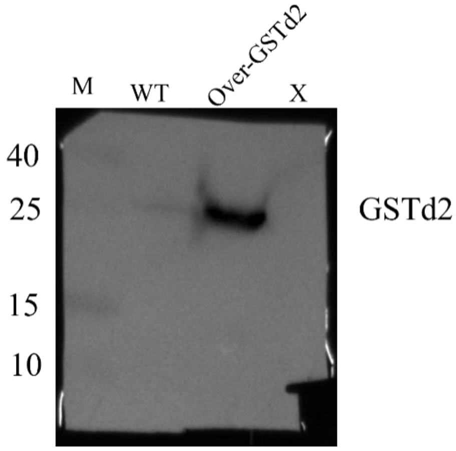

Protein level detection of GSTd2 in WT and Over-GSTd2 silkworms. WT: wild-type silkworms; Over-GSTd2: BmGSTd2 overexpression silkworms.

Figure 7D- tubulin gene

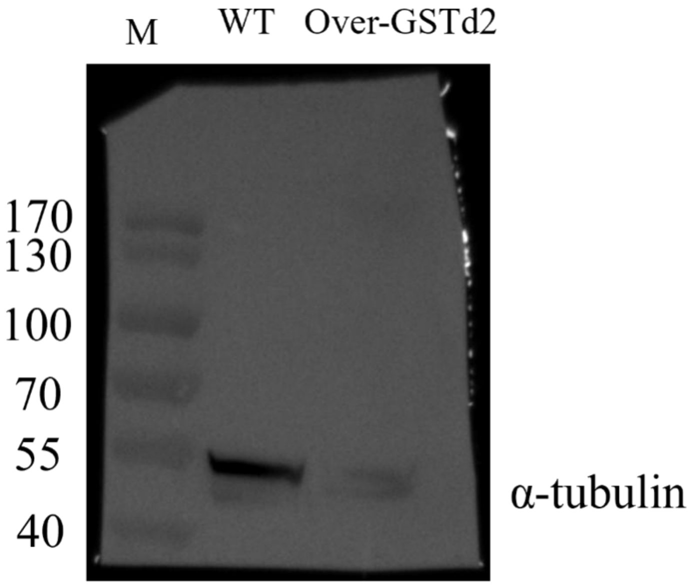

Protein level detection of α-tubulin in WT and Over-GSTd2 silkworms. WT: wild-type silkworms; Over-GSTd2: BmGSTd2 overexpression silkworms.

Sample	GSTd2(mean)	α-tubulin(mean)	Ratio (GSTd2/α-tubulin)
WT	1.065	13.160	0.080927
Over-GSTd2	25.186	2.354	10.699235

Quantitative Immunoblot Analysis of GSTd2 and α-tubulin in Over-expression individuals (Over-GSTd2) and WT individuals. GSTd2(mean): densitometric measurement of GSTd2 protein band intensity. α-tubulin(mean): densitometric

measurement of α-tubulin protein band intensity.
